# Editorial

**Published:** 1839-03-02

**Authors:** 


### THE MEDICAL EXAMINER.

PHILADELPHIA, MARCH 2, 1839.

A BILL is now before the Legislature of Pennsylvania, for the incorporation of a College of Physicians. It is proposed that the college shall have the power of granting degrees in medicine to candidates who may pass an examination upon such departments of science as are necessary for a physician, and who shall have pursued the course of study which may be directed by the college.

The proposed institution is to be very extended, and includes a very large number of physicians of Philadelphia; it differs from other associations of the kind, in one essential respect. The examiners are a board, appointed by the college, who do not teach; their interest is in no manner connected with the number of students who receive diplomas, for it is expressly understood that the fees which they may receive, are to be paid for the trouble and time which the examinations require, and are not to be refunded in case the candidate should be rejected. In other words, the ordinary mode of professional remuneration, founded upon the principle that the time of a physician is of value to him, is to be adopted. The lecturers and teachers are not to be selected from any body of men, nor need they belong to any one institution; all that is required is simply to produce to the college evidence that they really teach what they announce, and comply with the terms of their prospectus.

The student may, therefore, pursue his studies where he likes, and if he has reason to believe that he can gain more instruction from one teacher than from another, he may attend his class, and may not feel himself obliged to pursue the entire course of instruction required in the school to which the lecturer happens to be attached.

It is very plain, that if this system should be found practically to work well, it would obviate many of the disadvantages which are inherent in all institutions in which the teachers are the judges of the qualifications necessary for a degree; while, at the same time, it would in no wise interfere with the established schools. For in all cases, a school already established has a vantage ground, which, if well-conducted, it cannot be deprived of. This peculiarity of the new plan is one of its best features. We dislike the needless multiplication of schools, and we do not consider it just, that the exclusive privilege of granting degrees should be conferred upon any institution which may possess influence enough to obtain a charter. By the proposed system, the number of schools, that is, of associations of teachers, will be regulated by the usual laws of supply and demand, and physicians will very soon discover, that if a school which they form is not required, it will prove both a profitless and expensive undertaking. At the same time, a body of men or single individuals, who may possess the qualifications necessary for teachers, can always claim the right of bringing forward their pupils as candidates for examination, and will, therefore, be enabled to teach under conditions only a little less advantageous than those which are offered by established schools. The only important point of difference, is simply, the hold which older institutions have already obtained upon the affections of the public; this is a legitimate advantage, and one which we should regret to see endangered.

A plan similar to that which is now proposed, is obviously so much in harmony with the institutions of this country, that sooner or later it will undoubtedly be adopted. When nothing is asked of the government, except the authority permitting a body of competent individuals to grant an official certificate of the qualifications of pupils who may present themselves for examination, it will scarcely seem just to refuse the request. Concentration in a single school, or in a small number of schools, is impracticable in this country; and it is almost always found that new institutions succeed in obtaining separate charters; but the proposed bill may rather tend to diminish the number of future associations, and give greater scope to individual exertions.

In these remarks we have no allusion to existing schools. On the contrary, although we shall always remain untramelled as editors, we have our individual attachments; and our editorial efforts will be directed to uphold, and not to aid in breaking down, any institution which may gain for itself a desirable position, by the industry and ability of its teachers. We should advocate the proposed bill as one which seems well fitted to allay the heart-burnings and discontents of those who fancy themselves excluded from a legitimate career, while facilities will certainly be offered for instructers who may be well fitted for their task.

Much to the honour of the schools at Philadelphia, we understand that no objections are brought forward by their professors to a measure which, in all probability, will be, in the end, a means of increasing their usefulness and of strengthening their actual position.

